# Effects of matrix stiffness on epithelial to mesenchymal transition-like processes of endometrial epithelial cells: Implications for the pathogenesis of endometriosis

**DOI:** 10.1038/srep44616

**Published:** 2017-03-17

**Authors:** Sachiko Matsuzaki, Claude Darcha, Jean-Luc Pouly, Michel Canis

**Affiliations:** 1 CHU Clermont-Ferrand, CHU Estaing, Chirurgie Gynécologique, Clermont-Ferrand, France; 2 Clermont Université, Université d’Auvergne, ISIT UMR6284, Clermont-Ferrand, France; 3 CNRS, ISIT UMR6284, Clermont-Ferrand, France; 4 CHU Clermont-Ferrand, Service d’Anatomie et Cytologie Pathologiques, Clermont-Ferrand, France

## Abstract

Endometriosis is defined as the presence of endometrial glands and stroma within extrauterine sites. Our previous study revealed an epithelial to mesenchymal transition (EMT)-like process in red peritoneal endometriosis, whereas membrane localization of E-cadherin was well maintained in epithelial cells of deep infiltrating endometriosis (DIE). Here we show that endometrial epithelial cells (EEE) grown on polyacrylamide gel substrates (PGS) of 2 kilopascal (kPa), a soft matrix, initiate a partial EMT-like process with transforming growth factor-β1 (TGF-β1) stimulation. Increasing matrix stiffness with TGF-β1 stimulation reduced the number of cell-cell contacts. Cells that retained cell-cell contacts showed decreased expression of E-cadherin and zonula occludens 1 (ZO-1) to cell-cell junctions. Few deep endometriotic epithelial cells (DEE) grown on 30-kPa PGS, which may mimic *in vivo* tissue compliance of DIE, retained localization of E-cadherin to cell-cell junctions with TGF-β1 treatment. Immunohistochemical analysis showed no phosphorylated Smad 2/3 nuclear localization in E-cadherin+ epithelial cells of DIE. We hypothesize that EEE may undergo an EMT-like process after attachment of endometrium to peritoneum in a TGF-β1–rich microenvironment. However, TGF-β1 signaling may be absent in DIE, resulting in a more epithelial cell-like phenotype in a rigid microenvironment.

Endometriosis, a common gynecological disorder responsible for infertility and pelvic pain, is defined as the presence of endometrial glands and stroma within extrauterine sites^1^. This condition affects approximately 10% of women of reproductive age^1^. Despite extensive studies, the etiology, pathogenesis, and pathophysiology of endometriosis are not fully understood^1^. However, one of the most supported theories may be implantation theory: endometriosis originates from retrograde menstruation of endometrial tissue, which may then implant into the peritoneal cavity^1^.

Our previous study revealed epithelial to mesenchymal transition (EMT)- and mesenchymal to epithelial transition (MET)-like processes in epithelial cells of pelvic endometriosis^2^. We previously hypothesized that the origin of endometriotic epithelial cells might be endometrial epithelial cells^2^. Endometrial epithelial cells might be adapted to specific microenvironments after implantation, resulting in different types of pelvic endometriosis, including superficial peritoneal endometriosis, ovarian endometriosis, and deep infiltrating endometriosis (DIE)^2^. Endometrial epithelial cells might undergo an EMT-like process after attachment of endometrium to the peritoneum or ovary, resulting in red peritoneal endometriosis or ovarian endometriosis, respectively^2^. It has been postulated that red and black peritoneal lesions may represent different stages of the spontaneous evolution of endometriotic implants, with the first stage being red lesions^3^,^4^. MET-like processes may then occur during the evolution of peritoneal endometriotic implants, resulting in black peritoneal endometriosis^2^. E-cadherin expression is significantly higher in epithelial cells of DIE compared to those of menstrual endometrium, suggesting that a MET-like process might occur in DIE^2^. Black peritoneal lesions are generally much smaller than DIE tissue. We showed that expression levels of dephosphorylated beta-catenin were significantly higher in DIE compared to black peritoneal lesions^2^. The dephosphorylated form of beta-catenin is present at the plasma membrane upon Wnt stimulation^5^. Our previous *in vitro* study showed that the Wnt/beta-catenin pathway is involved in cell proliferation, migration, and/or invasion of endometriotic epithelial cells^6^. A more epithelial cell-like phenotype along with Wnt/beta-catenin pathway activation might facilitate growth and infiltration in DIE^2^, which is characterized histologically by dense fibrous tissue^3^,^7^. One of the hallmarks of fibrosis is tissue stiffening. The microenvironment of DIE is therefore more stiff than that of endometrium. Recent studies demonstrated that increased matrix stiffness could induce EMT^8^,^9^. A study showed that increasing matrix stiffness directly activated EMT through the EMT-inducing transcription factor TWIST1 in human MCF10A and tumorigenic mouse Eph4Ras mammary epithelial cells^8^. EMT can be induced or regulated by various growth and differentiation factors^10^,^11^. Among them, transforming growth factor-β1 (TGF-β1) may be a major inducer of physiological as well as pathological EMT during embryogenesis, cancer progression, and fibrosis^10^,^11^. TGF-β1 is also involved in the pathophysiology of endometriosis^12^.

These findings may not support our previous hypothesis^2^. However, to date, no study has investigated the effects of extracellular matrix (ECM) matrix stiffness on EMT-like processes in endometrial epithelial cells. The aim of the present *in vitro* study was to investigate the effects of ECM stiffness on EMT-like morphological and phenotypic changes of endometrial epithelial cells. Herein we used polyacrylamide gel substrates (PGS) of different stiffness (2-, 4-, 8-, 16-, and/or 30-kilopascal [kPa]) to evaluate the effects of substrate rigidity on expression of E-cadherin, zonula occludens 1 (ZO-1), N-cadherin, and F-actin in endometrial epithelial cells. We elected to use PGS of different degrees of stiffness based on the results of our previous study^13^. Jiang *et al*. showed that the elasticity of the endometrium was 3.34 ± 0.42 kPa during the proliferative phase and 1.97 ± 0.34 kPa during the secretory phase using three-dimensional multifrequency magnetic resonance elastography^14^. Currently no data are available regarding the tissue stiffness of DIE *in vivo*. However, several studies that evaluated fibrotic lungs, fibrotic intestines, and fibrotic livers have shown that the elastic modulus value ranged from approximately 1–3 kPa for normal tissue to approximately 17–22 kPa for fibrotic tissue^15^,^16^,^17^,^18^,^19^. The soft substrates (2- or 4-kPa PGS) and the rigid substrates (16- or 30-kPa PGS) may mimic *in vivo* tissue compliance of the endometrium or DIE, respectively^13^.

During EMT, epithelial cell-cell junctions, which are essential for epithelial integrity, are deconstructed, and the junction proteins are relocalized and/or degraded^11^,^20^,^21^. In cuboidal-shaped and epithelial cells organized in compact islets, F-actin is organized in cortical bundles tightly associated with cell-cell adhesions^11^,^20^,^21^. Cell morphology is changed to a spindle-shaped morphology and F-actin is assembled into contractile actin stress fibers across the ventral surface of the cells^11^,^20^,^21^. The dissolution of tight junctions during EMT is accompanied by the diffusion of ZO-1 from cell-cell contacts^11^,^20^,^21^. E-cadherin, a key component of adherens junctions, is cleaved at the plasma membrane and subsequently degraded^11^,^20^,^21^. Cadherin switching—the loss of E-cadherin and the gain of N-cadherin expression—is a major hallmark of EMT^11^,^20^,^21^.

## Results

### Effects of matrix stiffness with or without TGF-β1 treatment on E-cadherin, N-cadherin, ZO-1, and F-actin expression in endometrial epithelial cells of patients with endometriosis (EEE)

We first evaluated E cadherin expression in EEE (n = 5) and endometrial epithelial cells of patients without endometriosis (NEEE) (n = 5) grown on 2- or 30-kPa PGS, or plastic with or without TGF-β1 treatment. We observed no difference in E-cadherin expression between EEE and NEEE, when compared to cells grown on a substrate of the same stiffness (2- or 30-kPa PGS, or plastic). Thus, we further analyzed of E-cadherin, N-cadherin, ZO-1, and F-actin expression only in EEE.

#### Without TGF-β1 treatment

**Figure 1:**
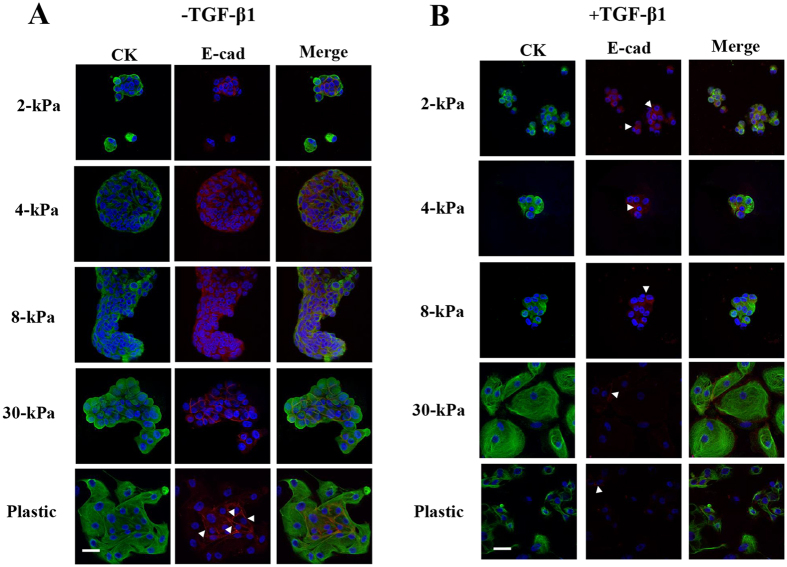
Representative photomicrographs of double-labeled immunofluorescence staining for CK and E-cadherin in EEE. (**A**) and (**B**): CK and E-cadherin in EEE grown on 2-, 4-, 8-, or 30-kPa polyacrylamide gel substrates (PGS), or on plastic without (**A**) or with (**B**) TGF-β1 (5 ng/mL) stimulation. (**A**): In EEE grown on 2-, 4-, 8-, or 30-kPa PGS, E-cadherin was localized to cell-cell junctions. In EEE grown on plastic, only cells located in the center retained localization of E-cadherin to cell-cell junctions (arrowheads). (**B**): Increasing matrix stiffness decreased E-cadherin localization to cell-cell junctions (arrowheads) Scale bar: 50 μm.

**Figure 2:**
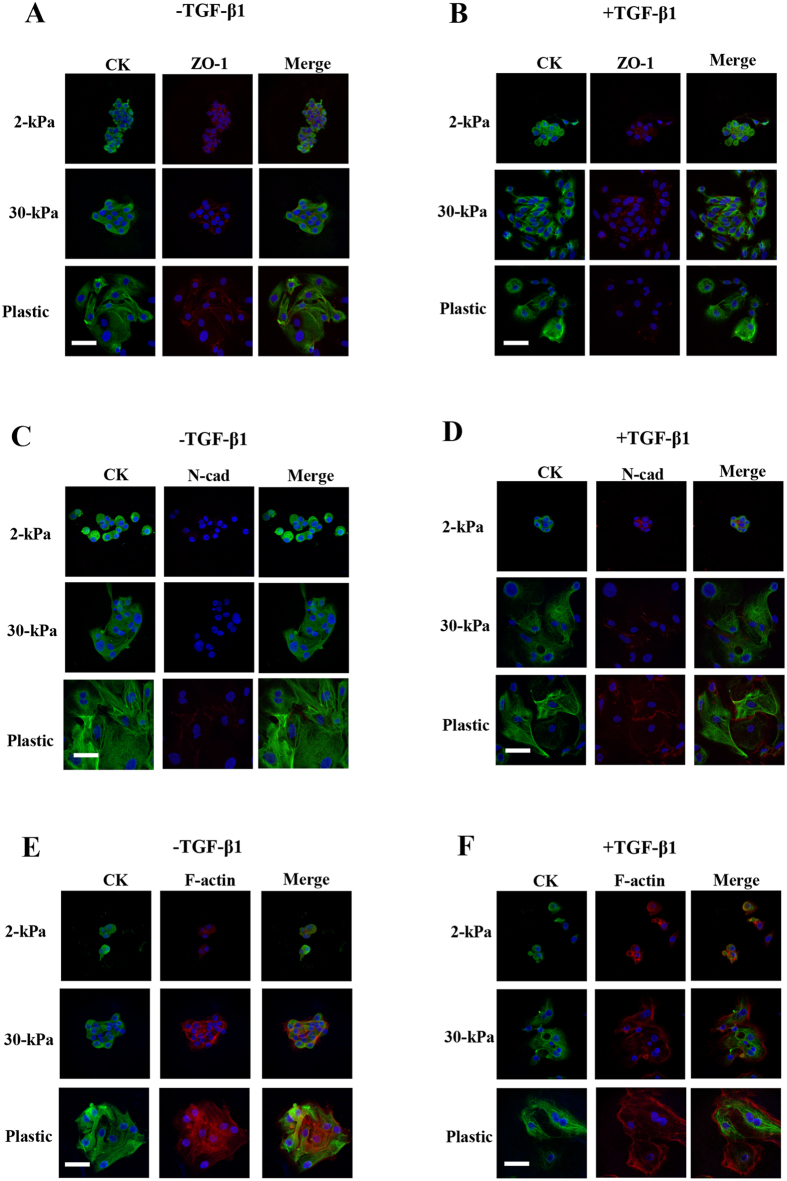
Representative photomicrographs of double-labeled immunofluorescence staining for CK and ZO-1, CK and N-cadherin and for CK and F-actin in EEE. (**A**,**B**) CK and ZO-1 in EEE grown on 2- or 30-kPa PGS, or on plastic without (**A**) or with (**B**) TGF-β1 (5 ng/mL) stimulation. (**C**,**D**) CK and N-cadherin in EEE grown on 2- or 30-kPa PGS, or on plastic without (**C**) or with (**D**) TGF-β1 (5 ng/mL) stimulation. (**E**,**F**) CK and F-actin in EEE grown on 2- or 30-kPa PGS or on plastic without (**E**) or with (**F**) TGF-β1 (5 ng/mL) stimulation. Scale bar: 50 μm.

EEE grown on 2-kPa PGS retained a rounded morphology, cortical actin, and E-cadherin and ZO-1 localization to cell-cell junctions (Figs 1A and 2A). In EEE grown on 2-, 4-, 8-, 16-, or 30-kPa PGS, cells retained cell-cell contacts. E-cadherin was localized to cell-cell junctions (Fig. 1A), but no N-cadherin localization to cell-cell junctions was observed (Fig. 2C). When EEE were grown on 30-kPa PGS, cells became elongated and F-actin+ stress fiber-like structures were observed (Fig. 2E). When EEE were grown on plastic, only cells located in the center retained retained localization of E-cadherin and ZO-1 to cell-cell junctions (Figs 1A and 2A). F-actin+ stress fiber-like structures were also observed (Fig. 2E). Cells were more loosely arranged than those grown on 30-kPa PGS (Figs 1A and 2A,C,E).

#### With TGF-β1 treatment

EEE grown on 2-kPa PGS largely retained an epithelial cobblestone morphology and cell-cell contact (Figs 1B and 2B). However, E-cadherin (Fig. 1B) and ZO-1 (Fig. 2B) localization to cell-cell junctions was decreased (Figs 1B and 2B), and localization of N-cadherin to cell-cell junctions, albeit in few cells, was observed (Fig. 2D). Peripheral actin filaments were also found around the cell body and protrusions (Fig. 2F). However, stress fibers were not found in the protrusions (Fig. 2F). Increasing matrix stiffness reduced the number of cell-cell contacts, and became elongated (Fig. 1B). Cells retained cell-cell contacts that were more loosely arranged, and decreased E-cadherin (Fig. 1B) and ZO-1 (Fig. 2B) and increased N-cadherin (Fig. 2D) localization to cell-cell junctions were observed. F-actin+ stress fiber-like structures were observed in EEE grown on 16- and 30-kPa PGS (Fig. 2F). When EEE were grown on plastic, morphological changes to fibroblast-like cells were observed in CK+ cells (Figs 1B and 2B,D,F). Few cells retained localization of E-cadherin and ZO-1 to cell-cell junctions (Figs 1B and 2B). The majority of cells were CK+ fibroblast-like single cells (Fig. 1B).

### Effects of matrix stiffness with or without TGF-β1 treatment on E-cadherin expression in endometriotic epithelial cells derived from DIE (DEE)

#### Without TGF-β1 treatment

**Figure 3:**
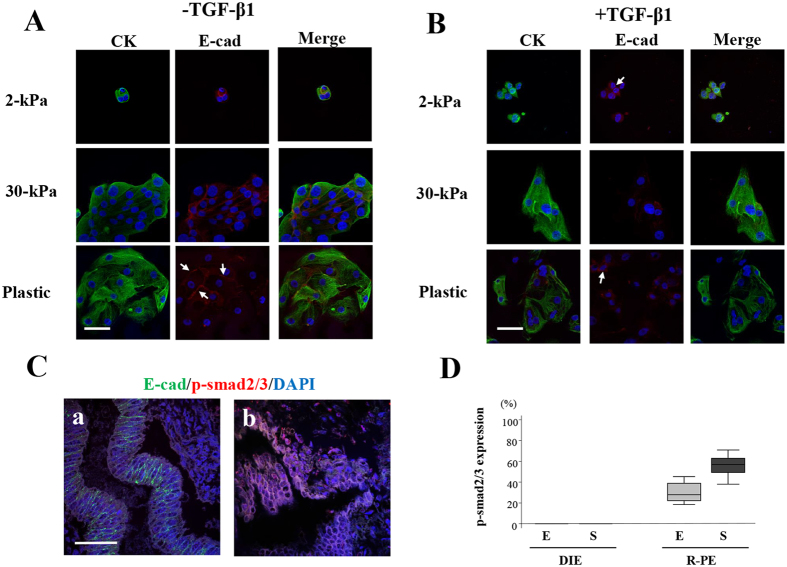
Representative photomicrographs of double immunofluorescence staining for CK and E-cadherin in endometriotic epithelial cells (DEE) derived from deep infiltrating endometriosis (DIE) and for E-cadherin and phosphorylated Smad 2/3 (p-Smad 2/3) in DIE and red peritoneal endometriotic lesions. (**A**,**B**) Double-labeled immunofluorescence staining for CK and E-cadherin in DEE grown on 2- or 30-kPa PGS or on plastic without (**A**) or with (**B**) TGF-β1 (5 ng/mL) stimulation. (**A**) In DEE grown on 2- or 30-kPa PGS, E-cadherin was localized to cell-cell junctions. In DEE grown on plastic, only cells located in the center retained localization of E-cadherin to cell-cell junctions (arrows). (**B**) Few cells retained localization of E-cadherin to cell-cell junctions (arrows). (**C**) Representative photomicrographs of double-labeled immunofluorescence staining for E-cadherin and p-Smad 2/3 in DIE (a) and red peritoneal endometriotic lesions (b). Scale bar: 50 μm. (**D**) p-Smad2/3 expression (percentage of p-Smad2/3+ nuclei among the total number of 4, 6-diamidino-2-phenylindole [DAPI]-stained nuclei) in DIE and red peritoneal endometriotic lesions (R-PE). E: epithelial cells. S: adjacent stromal cells to epithelial cells.

DEE grown on 2-kPa PGS retained a rounded morphology and E-cadherin localization to cell-cell junctions (Fig. 3A). When DEE were grown on 30-kPa PGS, cells became elongated, but retained E-cadherin localization to cell-cell junctions (Fig. 3A). DEE grown on plastic showed decreased E-cadherin localization to cell-cell junctions (Fig. 3A).

#### With TGF-β1 treatment

DEE grown on 2-kPa PGS largely retained an epithelial cobblestone morphology, but few cells retained localization of E-cadherin to cell-cell junctions (Fig. 3B). DEE grown on 30-kPa PGS had fewer cell-cell contacts and became elongated (Fig. 3B). Cells that retained cell-cell contacts were more loosely arranged as observed in EEE grown on 30-kPa PGS (Fig. 3B). Few cells retained localization of E-cadherin to cell-cell junctions (Fig. 3B).

When DEE was grown on plastic with TGF-β1 treatment, morphological changes to fibroblast-like cells were observed with TGF-β1 treatment (Fig. 3B). These fibroblast-like cells were CK+, but dissociated into single cells (Fig. 3B). Few cells retained localization of E-cadherin to cell-cell junctions (Fig. 3B).

### E-cadherin and phosphorylated Smad 2/3 (p-Smad 2/3) expression in DIE and red peritoneal endometriotic lesions

We selected five DIE samples with very high E-cadherin expression and five red peritoneal lesions with very low E-cadherin expression from samples analyzed in our previous study^2^. In these DIE tissues, no p-Smad 2/3 nuclear expression was observed (Fig. 3C,D). In contrast, nuclear p-Smad 2/3 expression was observed in both epithelial and stromal cells in red peritoneal lesions (Fig. 3C,D).

### Effects of matrix stiffness with or without TGF-β1 treatment on cell proliferation and Annexin V expression in EEE

**Figure 4:**
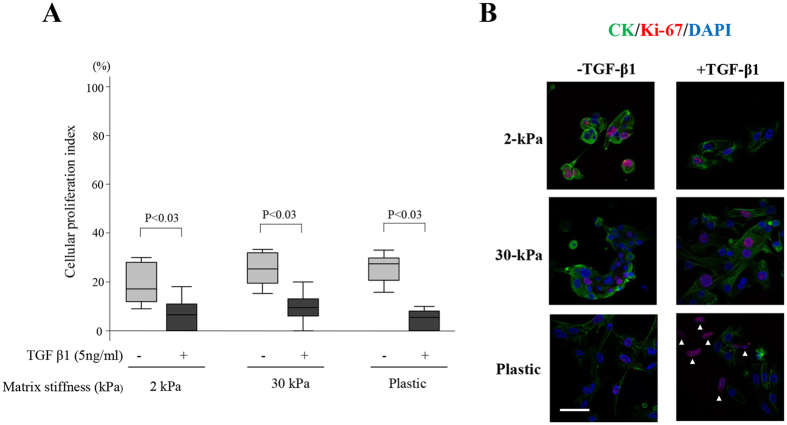
Effects of increasing matrix stiffness with or without transforming growth factor (TGF-β1) (5 ng/mL) stimulation on Ki-67 expression in endometrial epithelial cells of patients with endometriosis (EEE). (**A**) Cellular proliferation index (CPI) (percentage of Ki-67+ cells among the total number of cytokeratin [CK]+ cells) of proliferative EEE (n = 8) on PGS of varying stiffness (2- or 30-kPa) or on plastic. Numerical values are presented as box and whisker plots showing medians and the smallest and largest data point ≤1.5 × interquartile range (IQR) from the 25th and 75th percentiles, respectively. (**B**) Representative photomicrographs of Ki-67 and CK expression in EEE grown on 2- or 30-kPa substrates or plastic with or without TGF-β1 (5 ng/mL) stimulation. Arrowheads indicate of Ki-67+ /CK- cells. Scale bar: 50 μm.

No significant difference in the cellular proliferation index (CPI) was observed among cells grown on 2-kPa or 30-kPa PGS, or plastic, in cells treated with or without TGF-β1 (Fig. 4A,B). The CPI of EEE treated with TGF-β1 treatment was significantly lower than that without TGF-β1 treatment, when compared to cells grown on a substrate of the same stiffness (2- or 30-kPa, or plastic) (Fig. 4A,B). When cells were grown on plastic with TGF-β1 treatment, the majority of Ki-67+ cells (>90%) were CK- single cells (Fig. 4B).

No Annexin V+ cells were observed in cells grown on various substrates of stiffness (2- or 30-kPa, or plastic) with or without TGF-β1 treatment.

### Effects of increasing matrix stiffness and duration of cell culture on α-smooth muscle actin (αSMA)+ stress fibers and collagen type I protein expression in endometrial stromal cells of patients with endometriosis (EES)

**Figure 5:**
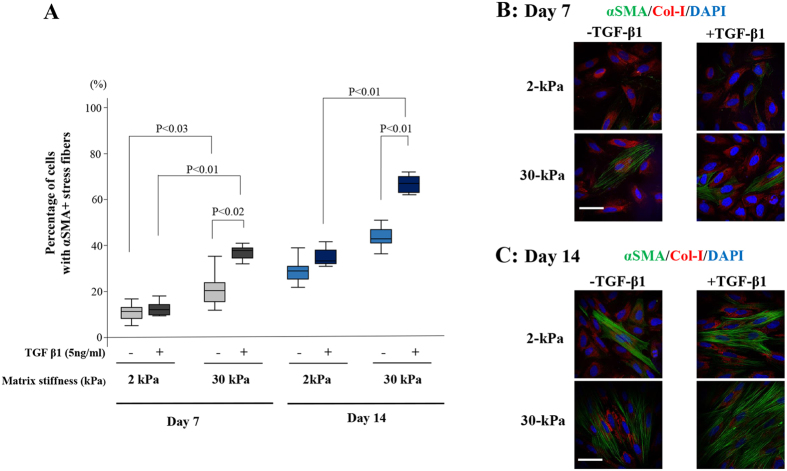
Effects of increasing matrix stiffness and the duration of cell culture on αSMA+ stress fibers and collagen type I protein expression in endometrial stromal cells of patients with endometriosis (EES). (**A**) The percentage of cells with αSMA+ stress fibers with or without TGF-β1 (5 ng/mL) stimulation in EES grown on 2- or 30-kPa PGS for 7 days or 14 days with or without TGF-β1 (5 ng/mL) stimulation. (**B**,**C**) Representative photomicrographs of αSMA+ stress fibers and collagen type I protein expression in EES grown on 2- or 30-kPa substrates for 7 days (**B**) or 14 days (**C**) with or without TGF-β1 (5 ng/mL) stimulation. Scale bar: 50 μm.

After 7- or 14-day cell culture, collagen type I protein expression was observed in all of the cells grown on 2-kPa or 30-kPa PGS with or without TGF-β1 treatment. The percentage of cells with αSMA+ stress fibers with or without TGF-β1 treatment was significantly increased in a time-dependent manner in both cells grown on 2-kPa and 30-kPa PGS (See Supplementary Fig. S1, Fig. 5B,C). TGF-β1 treatment significantly increased the percentage of cells with αSMA+ stress fibers in cells grown on 30-kPa PGS compared to those grown without TGF-β1 treatment (Fig. 5A,B,C). The percentage of cells with αSMA+ stress fibers was significantly higher in cells grown on 30-kPa PGS than those grown on 2-kPa PGS with or without TGF-β1 treatment after 7 days (Fig. 5A,B). However, after 14 days, no significant difference was observed in the percentage of cells with αSMA+ stress fibers grown on 2- and 30-kPa PGS without TGF-β1 treatment (Fig. 5A,C). With TGF-β1 treatment, the percentage of cells with αSMA+ stress fibers was significantly higher in cells grown on 30-kPa than those grown on 2-kPa PGS (Fig. 5A,C).

## Discussion

The present study showed that endometrial and endometriotic epithelial cells can sense changes in ECM stiffness and respond to them, resulting in morphological and phenotypic changes *in vitro*. We observed that endometrial epithelial cells as well as endometriotic epithelial cells derived from DIE, grown on plastic with or without TGF-β1 treatment, underwent a partial EMT-like process without full acquisition of mesenchymal characteristics. In the present study, we observed that the majority of cells grown on plastic with TGF-β1 treatment were single CK- cells. To date, no marker exists to distinguish CK- cells that are differentiated from CK+ epithelial cells through EMT from CK- fibroblasts. Thus, we cannot completely exclude the possibility that these CK- cells were derived from CK+ epithelial cells through complete EMT. However, endometrial stromal cells proliferated more on plastic than that on soft substrates^13^. In contrast, the present study showed no significant effect of matrix stiffness on cell proliferation of EEE. In the present study, we observed that the majority of Ki-67+ cells (>90%) was single CK- cells when they were grown on plastic with TGF-β1 treatment. Thus, we speculated that CK- cells may not be derived from CK+ endometrial epithelial cells through EMT, but rather may be endometrial stromal cells that were contaminated during isolation of endometrial epithelial cells. Findings from cells grown on plastic with a stiffness in the gigapascal (gPa) range, which is much stiffer than that occurring *in vivo* (kPa range), may not reflect the behavior of their *in vivo* counterparts. The present findings clearly showed that caution should be taken when interpreting an EMT-like process in primary endometrial or endometriotic epithelial cells grown on plastic or glass.

The present study showed that endometrial epithelial cells might begin to undergo a partial EMT-like process even on 2-kPa PGS, a soft matrix, when cells are stimulated with TGF-β1. A previous study showed that decreasing matrix stiffness increased TGF-β1–induced apoptosis in normal murine mammary gland epithelial cells (NMuMG) and Madin-Darby canine kidney epithelial cells (MDCK)^9^. However, in the present study, we observed no Annexin V+ EEEs grown on a soft matrix with TGF-β1 treatment and showed that cell proliferation was similar to that of cells grown on a rigid matrix.

A recent study showed that the peritoneal mesothelium may be responsible for the increased TGF-β1 levels in women with endometriosis^22^. The study investigators speculated that the development of peritoneal endometriosis and the increase in TGF-β1 are likely to go hand-in-hand, because retrograde menstruation and the presence of endometrial cells within the peritoneal cavity can induce inflammation^22^. If the origins of endometriotic epithelial cells are endometrial epithelial cells, endometrial epithelial cells undergo an EMT-like process after implantation into the peritoneum, resulting in red peritoneal lesions in a TGF-β1 rich microenvironment. A limitation of the present study, however, is that endometrial epithelial cells derived from the menstrual phase were collected from only a limited number of patients. Most endometrial epithelial cell samples were derived from the proliferative phase. If the origins of endometriotic epithelial cells are endometrial epithelial cells as stated by implantation theory, endometrial epithelial cells derived from the menstrual phase would be more appropriate for investigation.

The present study showed that few endometriotic epithelial cells derived from DIE retained localization of E-cadherin to cell-cell junctions, when cells were grown on 30-kPa PGS, a rigid substrate, with TGF-β1 treatment. In view of the present results and our previous findings^2^, we speculated that TGF-β1 signaling may be absent in DIE that maintains E-cadherin expression *in vivo*^2^. Two studies have used intravital imaging to show that TGF-β1 signaling is transiently and locally activated in disseminating single cells, whereas cancer cells migrate collectively in the absence of TGF-β1 signaling *in vivo*^23^,^24^. Collectively migrating cells overexpressed epithelial biomarkers, and cadherin-mediated cell-cell adhesions were crucial for cell-cell coordination during collective migration^25^,^26^. Increasing substrate stiffness increased collective cell migration speed^27^. Membrane localization of E-cadherin as well as ZO-1 was maintained in clusters of TbRII KO tumors^24^, whereas neither was maintained in TbRIIfl/fl tumors at the tumor-stromal interface^24^. A previous experimental study in a baboon model of endometriosis suggested that collective migration may be involved in pathophysiological processes of DIE^28^. In addition, cancer cells with high levels of TGF-β1 signaling failed to promote lung metastasis, caused by failure of cells to proliferate in the lungs^23^. The downregulation of TGF-β1 signaling at metastatic sites then permits growth of metastatic tumors^23^. The loss of TGF-β1 signaling was significantly correlated with increased tumor size and enhanced carcinoma cell survival^23^. DIE tissue is generally much bigger in size than red peritoneal endometriotic lesions. A previous immunohistochemical study showed that expression of a marker of active TGF-β1 signaling, p-Smad 2, was most pronounced in endometriotic epithelial cells of DIE^29^. However, it was not clear whether p-Smad 2 was localized in E-cadherin+ endometriotic epithelial cells of DIE. Thus, we further performed double immunofluorescence staining for E-cadherin and p-Smad 2/3 in endometriotic tissues of DIE and red peritoneal lesions. These findings may support our speculation that TGF-β1 signaling may be absent in DIE. However, a limitation of the present study is a tremendous gap between *in vivo* tissue findings by immunohistochemical analysis and the present *in vitro* experiments. In addition, the absence of TGF-β1 signaling in already developed surgically excised DIE does not indicate that it had been absent during development of DIE. As speculated for cancer^23^,^24^, it is more likely that TGF-β1 signaling is transiently and locally activated during the development of DIE. Further experiments are required to confirm our speculation.

However, if TGF-β1 signaling is absent throughout DIE development, how do fibrotic microenvironments in DIE develop? TGF-β1 is the most potent key mediator of fibrosis^30^. Our previous studies also supported the importance of the TGF-β1 signaling in fibrosis of endometriosis^13^,^31^,^32^ and suggested that TGF-β1 induces ECM synthesis and remodeling, as well as myofibroblast differentiation^13^,^31^,^32^. Moreover, evidence suggests that TGF-β1 is involved in the pathophysiology of endometriosis^12^. Our previous study revealed that αSMA+ stress fibers in very few or no EES grown on 30-kPa PGS with 72-h TGF-β1 stimulation^13^. However, in the present study, we showed that a longer culture duration promoted EES to differentiate into myofibroblasts without TGF-β1 treatment. In addition, not only myofibroblast cells produce collagen type I: after implantation, EES may differentiate into myofibroblasts and produce collagen type I; increased stiffness through increased myofibroblast collagen production may then further increase matrix stiffness, resulting in a fibrotic microenvironment in DIE over time. Thus, the present findings suggest that TGF-β1 signaling may not be indispensable to the development of the fibrotic microenvironment in DIE.

In the present study, only epithelial or stromal cells were cultured, but endometriotic tissue and endometrium are composed of multiple cell types and extracellular matrix. Cell culture systems that more closely mimic the cellular complexity typical of *in vivo* tissues are required to investigate whether and how TGF-β1 signaling pathway is involved in the pathophysiology of different types of pelvic endometriosisin. Such investigations could provide important information to support the development of novel therapeutic strategies for endometriosis.

In conclusion, the present studies showed that cells retain the epithelial-related phenotype in EEE grown on substrates of various stiffness (2-, 4-, 8-, 16-, and/or 30-kPa), when cells are not stimulated with TGF-β1. However, EEE might begin to undergo a partial EMT-like process even on a soft matrix (2-kPa), when cells are stimulated with TGF-β1. We hypothesize that EEE may undergo an EMT-like process after attachment of endometrium to peritoneum in a TGF-β1–rich microenvironment. However, TGF-β1 signaling may be absent in DIE, resulting in a more epithelial cell-like phenotype in a rigid microenvironment.

## Materials and Methods

### Patients

Patients age 20–37 years undergoing laparoscopy for endometriosis were recruited at CHU Clermont-Ferrand, France for the present study. None of the women had received hormonal therapy and none used intrauterine contraception for at least 6 months prior to surgery. Recruited patients had regular menstrual cycles (26–32 days) with confirmation of their menstrual history. Endometrial samples from 40 patients who had histological evidence of DIE and DIE samples from 5 patients were used for the present *in vitro* analysis. In addition, endometrial tissues from 5 patients without endometriosis (patients with uterine fibroma: n = 2, patients with tubal infertility: n = 3) were obtained. The clinical characteristics of patients are shown in Supplementary Table S1. The numbers of samples used for each experiment are summarized in Supplementary Table S2.

The research protocol was approved by the Consultative Committee for Protection of Persons in Biomedical Research (CPP) of the Auvergne (France) region. All experiments were performed in accordance with the approved guidelines and regulations. Informed written consent was obtained from each patient prior to tissue collection.

### Cell culture

Endometrial and endometriotic epithelial cells and endometrial stromal cells were isolated as previously described^6^. Isolated cells were plated onto Primaria flasks (BD, Le Pont-De-Claix, France) in phenol red-free Dulbecco’s modified Eagle medium (DMEM)/F-12 (Life Technologies, Cergy Pontoise, France) containing 10% charcoal-stripped fetal bovine serum (FBS) (Sigma-Aldrich, Lyon, France), 100 U/mL penicillin (Sigma-Aldrich), 0.1 mg/mL streptomycin (Sigma-Aldrich), and 0.25 μg/mL amphotericin B (Sigma-Aldrich) and incubated at 37 °C in 95% air/5% CO_2_. Epithelial cells were incubated at 37 °C in 95% air/5% CO_2_ for 60 min to allow contaminated stromal cells to attach to the flask wall. The nonattached endometrial or endometriotic epithelial cells were recovered and used, whereas endometrial stromal cells at passage 1 were used for the present experiments. Immunofluorescence staining was performed to determine the purity of the isolated epithelial and stromal cells as previously described^6^,^13^. The results indicated that the purity of epithelial and stromal cells was >98% and >99%, respectively.

### Preparation of polyacrylamide gel supports

Polyacrylamide gels of variable stiffness were prepared on glass coverslips using modifications to the protocol of Fischer *et al*.^33^ as previously described^13^. The polyacrylamide gel can be maintained for several days. For a longer cell culture of endometrial stromal cells, stiffness-controlled 96-well plates were prepared using modifications to the protocol of Syed *et al*.^34^.

Cells were seeded onto coated gels or plastic in full growth medium (phenol red-free DMEM/F-12 containing 10% charcoal-stripped FBS, 100 U/mL penicillin, 0.1 mg/mL streptomycin, and 0.25 μg/mL amphotericin B) (Life Technologies) and incubated for 2–3 h at 37 °C in 95% air/5% CO_2_ to allow adherence. The supernatant medium was removed and the desired cell culture medium (2% charcoal-stripped FBS with or without TGF-β1 [5 ng/mL]) (R&D Systems, Lille, France) was overlaid.

Then, endometrial epithelial cells were cultured for 120 h with or without TGF-β1 on 2-, 4-, 8-, 16-, or 30-kPa PGS, or plastic. Endometriotic epithelial cells derived from DIE were cultured for 120 h with or without TGF-β1 on 2- or 30-kPa PGS, or plastic. Endometrial stromal cells were cultured for 7 days or 14 days with or without TGF-β1 on 2- or 30-kPa PGS.

A major disadvantage of polyacrylamide is its cytotoxicity. However, this is not a concern for the present 2D culture models in which cells are seeded on top of polyacrylamide gels^35^.

### Immunofluorescence staining

Double immunofluorescence staining of endometrial and endometriotic epithelial cells and endometrial stromal cells was performed according to the protocol published by Lee *et al*.^36^. In endometrial epithelial cells, double immunofluorescence staining for Ki-67 (D2H10, 1;200, Cell Signaling, Danvers, MA, USA) and cytokeratin (CK) (MNF116, 1:100, DAKO, Glostrup, Denmark), F-actin (Alexa Fluor 594-phalloidin, Life Technologies)/CK, E-cadherin (4A2, 1:50, Cell Signaling)/CK ZO-1 (D6L1E, 1:400, Cell Signaling)/CK, and N-cadherin (D4R1H, 1:200, Cell Signaling)/CK was performed. In endometriotic epithelial cells, double immunofluorescence staining for E-cadherin/CK was performed. In endometrial stromal cells, double immunofluorescence staining for collagen I (rabbit polyclonal, 1:500, Abcam, Cambridge, UK) and αSMA (1A4, 1:100, Merck Millipore) was performed.

In addition, double immunofluorescence staining on paraffin sections for E-cadherin (NCH38, 1:50, DAKO) and p-Smad 2/3 (rabbit polyclonal, 1:200, Santa Cruz Biotechnology, Santa Cruz, CA, USA) was performed in deep endometriotic and red peritoneal endometriotic tissues. Sections were deparaffinized, and antigen retrieval was performed; sections were then treated with 3% H_2_O_2_ solution as described previously^6^. Sections were incubated overnight at 4 °C with primary antibodies against E-cadherin and p-Smad 2/3.

Alexa Fluor 488 (green) goat anti-mouse IgG and Alexa Fluor 594 (red) goat anti-rabbit IgG conjugated antibodies (Life Technologies) were used as secondary antibodies. Cell nuclei were stained with 4, 6-diamidino-2-phenylindole (DAPI) (Life Technologies). Slides were analyzed with a Leica TCS SPE confocal laser-scanning microscope (Leica Microsystems, Nanterre, France). The cellular proliferation index (CPI) (percentage of Ki-67+ cells among the total number of CK+ cells), the percentage of cells with αSMA+ stress fibers, and the percentage of collagen I+ cells among the total number of DAPI-stained nuclei, were calculated from 10 random high-power (x400) fields through each section. For p-Smad2/3 nuclear expression, the percentage of p-Smad2/3+ nuclei among the total number of DAPI-stained nuclei was calculated from the entire field of each section.

### Analysis of apoptosis by Annexin V staining

Cells grown on 2-kPa or 30-kPa PGS or plastic for 120 h with or without TGF-β1 were stained with Annexin V-FITC (Annexin V kit, Beckman Coulter, Villepinte, France) according to the manufacturer’s protocol. Cell nuclei were stained with DAPI (Life Technologies). Slides were analyzed with a Leica TCS SPE confocal laser-scanning microscope (Leica Microsystems).

### Statistical analysis

The STATA program version 12 (StataCorp, College Station, TX, USA) was used for statistical analysis. Comparisons between different groups were made using the Wilcoxon matched pairs signed-ranks test or the Mann-Whitney *U* test. Statistical significance was defined as p < 0.05.

## Additional Information

**How to cite this article**: Matsuzaki, S. *et al*. Effects of matrix stiffness on epithelial to mesenchymal transition-like processes of endometrial epithelial cells: Implications for the pathogenesis of endometriosis. *Sci. Rep.* **7**, 44616; doi: 10.1038/srep44616 (2017).

**Publisher's note:** Springer Nature remains neutral with regard to jurisdictional claims in published maps and institutional affiliations.

## Supplementary Material

Supplementary Information
