# Supplementary material for: Effects of matrix stiffness on epithelial to mesenchymal transition-like processes of endometrial epithelial cells: Implications for the pathogenesis of endometriosis
Source: Sci Rep. 2017 Mar 17;7:44616. doi: 10.1038/srep44616 (PMC5356009; doi:10.1038/srep44616)
Supplement: Supplementary Information [file srep44616-s1.pdf]

Effects of matrix stiffness on epithelial to mesenchymal transition-like processes of endometrial epithelial cells: Implications for the pathogenesis of endometriosis

Sachiko Matsuzaki 1, 2, 3*

Claude Darcha 4

Jean-Luc Pouly 1

Michel Canis 1, 2, 3

1 CHU Clermont-Ferrand, CHU Estaing, Chirurgie Gynécologique, Clermont-Ferrand, France

2 Clermont Université, Université d'Auvergne, ISIT UMR6284, Clermont-Ferrand, France

3 CNRS, ISIT UMR6284, Clermont-Ferrand, France

4 CHU Clermont-Ferrand, Service d'Anatomie et Cytologie Pathologiques, Clermont-Ferrand, France

*To whom correspondence should be addressed:

Sachiko Matsuzaki

CHU Clermont-Ferrand, CHU Estaing, Chirurgie Gynécologique, 1, Place Lucie et Raymond Aubrac, 63003 Clermont-Ferrand, France, Tel: +33 4 73 75 01 38, E-mail: sachikoma@aol.com

Supplementary Figure 1

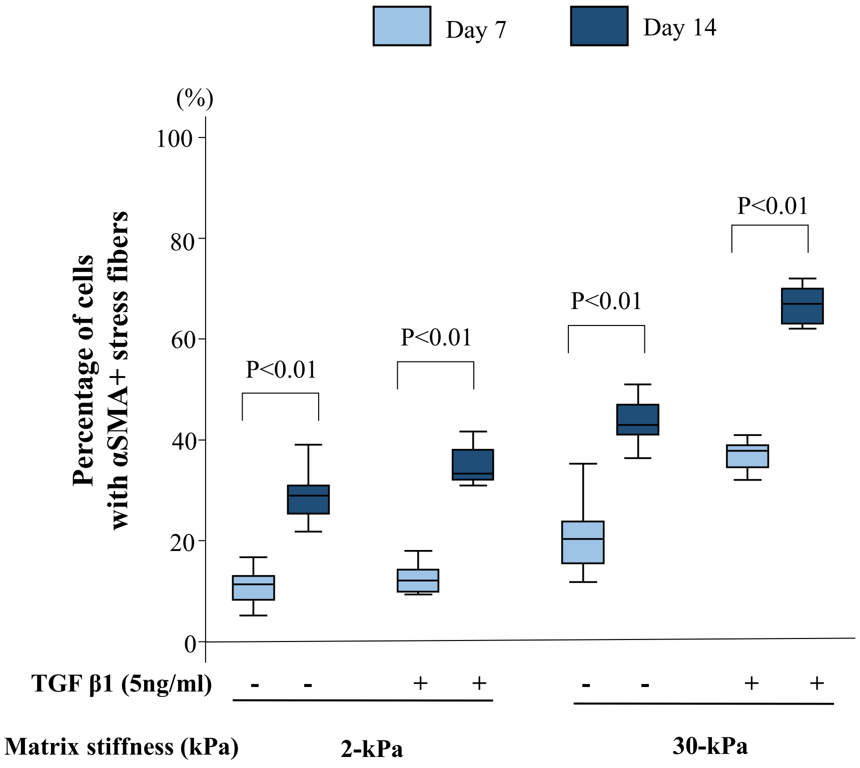

Supplementary Figure Legends

Supplementary Figure 1: The percentage of cells with αSMA+ stress fibers with or without TGF-β1 (5 ng/mL) stimulation in endometrial stromal cells of patients with endometriosis (EES) grown on 2- or 30-kPa PGS for 7 days or 14 days with or without TGF-β1 (5 ng/mL) stimulation.

Supplementary Table 1. Clinical characteristics of patients

	With Endometriosis	Without endometriosis
No of cases	40	5
Age [a]	31.0 (23-37)	30.0 (24-35)
Parity [a]	0 (0-1)	0 (0-1)
rASRM stage[b]		
I	18	
II	12	
III	4	
IV	6	

[a] Median (range)

[b] Revised American Society for Reproductive Medicine classification (rASRM) (American Society for Reproductive Medicine, 1997).

Supplementary Table 2: The numbers of samples used for each experiment

	EEE	NEEE	DEE	EES	DIE	R-PE
CK & E-cadherin	10	5	5			
CK & N-cadherin	6					
CK & ZO-1	6					
CK & F-actin	6					
E-cadherin & p-Smad 2/3					5	5
CK & Ki67	8					
Annexin V staining	8					
αSMA & Col-I				8		

EEE: endometrial epithelial cells of patients with endometriosis

NEEE: endometrial epithelial cells of patients without endometriosis

DEE: deep infiltrating endometriotic epithelial cells

EES: endometrial stromal cells of patients with endometriosis

DIE: deep infiltrating endometriotic tissues

R-PE: red peritoneal endometriotic tissues

CK: cytokeratin

ZO-1: zonula occludens 1

p-Smad 2/3: phosphorylated Smad 2/3

αSMA: α-smooth muscle actin

Col-I: collagen I
